# A p38MAPK/MK2 signaling pathway leading to redox stress, cell death and ischemia/reperfusion injury

**DOI:** 10.1186/1478-811X-12-6

**Published:** 2014-01-14

**Authors:** Muhammad Imtiaz Ashraf, Matthias Ebner, Christoph Wallner, Martina Haller, Sana Khalid, Hubert Schwelberger, Katarzyna Koziel, Marion Enthammer, Martin Hermann, Stephan Sickinger, Afschin Soleiman, Christina Steger, Stephanie Vallant, Robert Sucher, Gerald Brandacher, Peter Santer, Duska Dragun, Jakob Troppmair

**Affiliations:** 1 Daniel Swarovski Research Laboratory, Department of Visceral, Transplant- and Thoracic Surgery, Innsbruck Medical University (IMU), Innrain 66, 6020 Innsbruck, Austria; 2 Molecular Biology Laboratory, Department of Visceral, Transplant and Thoracic Surgery, Innsbruck Medical University (IMU), Schöpfstraße 41, Innsbruck 6020 Austria; 3 Department of Anesthesiology and Critical Care Medicine, Innsbruck Medical University (IMU), Anichstraße 35, Innsbruck, 6020, Austria; 4 Department of Pediatrics II, Innsbruck Medical University, Anichstraße 35, 6020 Innsbruck, Austria; 5 Soleiman Pathologie, Kugelanger 12a, 8060 Hall in Tirol, Austria; 6 Department for Pathology, Innsbruck Medical University, Innsbruck Medical University (IMU), Anichstraße 35, 6020 Innsbruck Austria; 7 Clinic for Nephrology and Intensive Care Medicine, Charité, Augustenburger Platz 1, Berlin, 13353 Germany; 8 Current address: Reconstructive Transplantation Laboratory, Department of Plastic and Reconstructive Surgery, Johns Hopkins University School of Medicine, Baltimore, MD USA; 9 Current address: Department of Anesthesia, Critical Care and Pain Medicine, Massachusetts General Hospital, Harvard Medical School, Boston, MA USA

**Keywords:** p38MAPK signaling, Ischemia/reperfusion injury (IRI), Reactive oxygen species (ROS), Apoptosis, Kidney

## Abstract

**Background:**

Many diseases and pathological conditions are characterized by transient or constitutive overproduction of reactive oxygen species (ROS). ROS are causal for ischemia/reperfusion (IR)-associated tissue injury (IRI), a major contributor to organ dysfunction or failure. Preventing IRI with antioxidants failed in the clinic, most likely due to the difficulty to timely and efficiently target them to the site of ROS production and action. IR is also characterized by changes in the activity of intracellular signaling molecules including the stress kinase p38MAPK. While ROS can cause the activation of p38MAPK, we recently obtained *in vitro* evidence that p38MAPK activation is responsible for elevated mitochondrial ROS levels, thus suggesting a role for p38MAPK upstream of ROS and their damaging effects.

**Results:**

Here we identified p38MAPKα as the predominantly expressed isoform in HL-1 cardiomyocytes and siRNA-mediated knockdown demonstrated the pro-oxidant role of p38MAPKα signaling. Moreover, the knockout of the p38MAPK effector MAPKAP kinase 2 (MK2) reproduced the effect of inhibiting or knocking down p38MAPK. To translate these findings into a setting closer to the clinic a stringent kidney clamping model was used. p38MAPK activity increased upon reperfusion and p38MAPK inhibition by the inhibitor BIRB796 almost completely prevented severe functional impairment caused by IR. Histological and molecular analyses showed that protection resulted from decreased redox stress and apoptotic cell death.

**Conclusions:**

These data highlight a novel and important mechanism for p38MAPK to cause IRI and suggest it as a potential therapeutic target for prevention of tissue injury.

## Background

Ischemia/reperfusion injury (IRI) contributes to morbidity and mortality in a wide range of pathologies including acute coronary syndrome, stroke, acute kidney injury, sickle cell disease and is particularly unavoidable during solid organ transplantation [1]. ROS are central to the initiation and progression of damage to organs throughout ischemia/reperfusion (IR). In particular, during the early phase of reperfusion excessive amounts of ROS are produced, which through direct damage to biomolecules or indirectly through the activation of intracellular signaling pathways cause progression of the injury. ROS have been linked to inflammasome activation [2,3] and autophagy [4,5], and once produced they thus have important pacemaker function on the path to full-blown IRI. Both, mitochondrial and non-mitochondrial (i.e. NADPH-dependent oxidases, NOXs) ROS producing systems are involved in IRI. Strikingly, eliminating a single system already resulted in a therapeutic benefit. Thus genetic ablation of the mitochondrial ROS producing enzyme p66SHC [6] or the inhibition of complex I of the mitochondrial electron transport chain (ETC), a major source for ROS, through S-nitrosation [7] prevented IRI in the heart, which could also be achieved by the knockout of NOX2 or NOX4 [8]. Ablation of both NOX isoforms exacerbated IRI, stressing the importance of maintaining basal ROS signaling [8].

Despite the acknowledged role of ROS, the use of anti-oxidants failed to yield a convincing therapeutic benefit [9]. ROS are short-lived and highly reactive and thus much of the damage happens rapidly and in close proximity to the site of production and scavenging mitochondrial ROS is thus technically challenging. Most desirable, therefore, are strategies to prevent excessive ROS generation. Intracellular signaling pathways are increasingly realized for the control they can exert over mitochondria. Oxidative phosphorylation (OXPHOS), a main source of mitochondrial ROS, depends on respiratory super complexes in mitochondria [10], whose reversible phosphorylation and other forms of posttranslational modifications provide important layers of regulation [11]. This is best understood for protein kinase A (PKA) that phosphorylates components of the mitochondrial electron transport chain (ETC), and thereby decreases ROS production under cellular stress [12]. Activation of intracellular signaling molecules including mitogen-activated protein kinases (MAPKs) during IR is well documented [13,14]. MAPK comprise a family of related kinases, which function downstream of similarly evolutionary conserved upstream signaling components [15]. The initially characterized signaling proteins ERK1, 2 are targets of mitogenic signaling downstream of growth factor receptors, RAS, RAF and MEK [16,17], while the related MAPKs p38MAPK and JNK, also referred to as stress kinases, serve important functions in cytokine signaling but also cell death induction [15]. In various cell and organ systems p38MAPK activity is increased upon reoxygenation/reperfusion and we recently provided first evidence that its activity may be linked to ROS generation. These ROS were also essential for cell death induction *in vitro*[14] (and unpublished data), a major consequence of p38MAPK signaling during IR [14,18-21]. To confirm p38MAPK as inducer of ROS-initiated damage to cells and organs, we used two experimental approaches, hypoxia/reoxygenation (HR) *in vitro* on HL-1 cardiomyocytes and mouse embryonic fibroblasts (MEFs) and kidney clamping in the rat, a well established model for the study of ischemia/reperfusion injury (IRI) *in vivo*.

## Results

### p38MAPKα regulates mitochondrial ROS accumulation during hypoxia/reoxygenation (HR)

**Figure 1:**
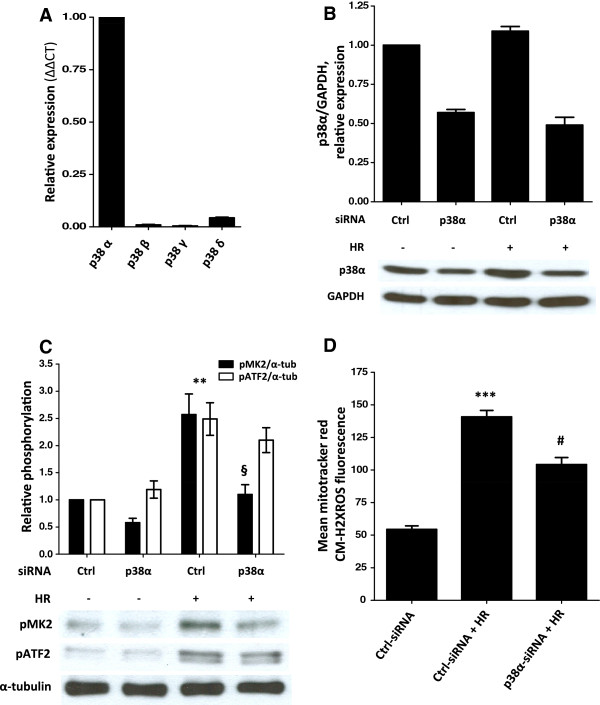
**Knockdown of p38MAPKα (p38α) decreases ROS levels following HR. (A)** Quantitative RT-PCR analysis of p38MAPK isoform expression in HL-1 cells (n = 3). **(B-D)** Effect of p38MAPKα knockdown on downstream signaling and mitochondrial ROS production. 72 hours after transfection with p38MAPKα siRNAs (250 nM) or control siRNAs (250 nM), HL-1 cells were exposed to the following HR protocol: hypoxia (1 hour) and reoxygenation (15 min) and analyzed for the expression of p38MAPKα **(B)**, phosphorylation of MK2 and ATF2 **(C)** and mitochondrial ROS levels **(D)** as described in Methods. Representative immunoblots and summary graphs are shown **(B-D)**. The data are expressed as mean ± SEM (n = 3-4). **p < 0.01, ***p < 0.001 control siRNAs transfected cells vs. control siRNA transfected cells undergoing HR; §*p* < 0.01, #*p* < 0.001 control siRNA transfected HL-1 cells vs. p38MAPKα siRNA transfected cells, subjected to HR.

We have shown previously that ischemia in a heterotopic heart transplant model and hypoxia in cardiomyocytes *in vitro* increased p38MAPK activity, which was further enhanced during reperfusion and reoxygenation, respectively [14]. Strikingly, p38MAPK inhibition reduced mitochondrial ROS levels and prevented cell death [14]. To corroborate these findings we first established the expression pattern of p38MAPK isoforms in HL-1 cells by quantitative real time PCR. This work identified p38MAPKα as the predominantly expressed isoform in these cells (Figure 1A). These results were also confirmed at the protein level (data not shown). To substantiate the involvement of p38MAPKα in regulating mitochondrial ROS levels under cellular stress siRNAs were used to decrease p38MAPKα expression (Figure 1B). We observed activation of p38MAPK during HR as monitored by the phosphorylation of its substrates MAPKAP kinase 2 (MK2) [22] and activating transcription factor-2 (ATF2) (Figure 1C). MK2 phosphorylation was significantly reduced following downregulation of p38MAPKα, however, the phosphorylation of the other p38MAPK substrate tested, ATF2 [22], was not affected (Figure 1C), suggesting alternative pathways for activating ATF2. As reported previously [14], HR resulted in increased ROS levels in HL-1 cells, which were significantly decreased in cells transfected with siRNAs against p38MAPKα (Figure 1D).

### Role of MAPKAP kinase 2 (MK2) in signaling downstream of p38MAPK

**Figure 2:**
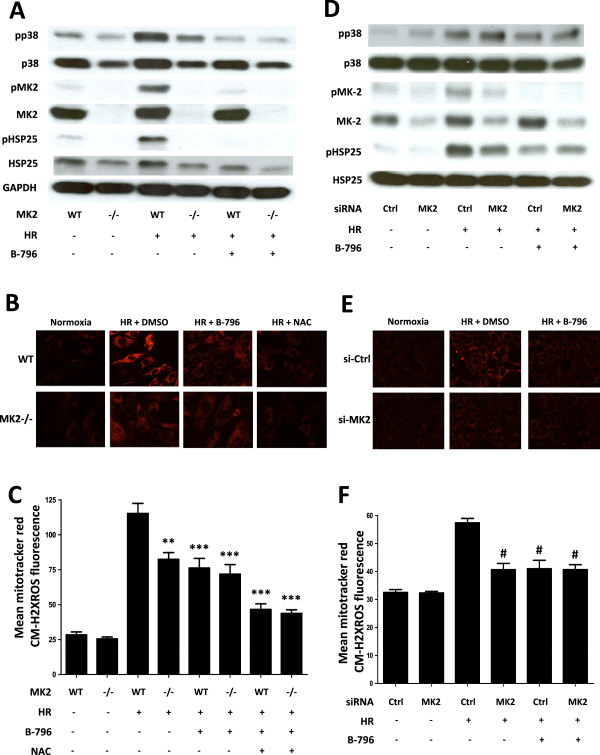
**p38MAPK (p38) increases mitochondrial ROS levels via MK2. (A)** WT and MK2-/- MEFs were pretreated with vehicle or BIRB796 (B-796) (50 nM) for 1 hour and then subjected to HR: hypoxia (6 hours) and reoxygenation (15 min). Expression of phosphorylated and non-phosphorylated p38MAPK, MK2 and HSP25 was determined. **(B, C)** For mitochondrial ROS measurements WT and MK2-/- MEFs were pretreated with either vehicle, BIRB796 (B-796) (50 nM) or N-acetyl cysteine (NAC) (7.5 mM) for 1 hour and exposed to the HR protocol followed by ROS measurement as described in Methods. **(D-F)** 72 hours after transfection with MK2 siRNAs (500 nM) or control siRNAs (500 nM), HL-1 cells were subjected to HR: hypoxia (6 hours) and reoxygenation (15 min) and analyzed for the effect of MK2 knockdown on p38MAPK/MK2 signaling **(D)**, and mitochondrial ROS production **(E, F)** during HR as described in the Methods. Representative immunoblots **(A, D)**, fluorescence images **(B, E)** and summary graphs **(C, F)** are shown. The data are expressed as mean ± SEM (n = 6-8). **p < 0.01, ***p < 0.001 vs. WT MEFs undergoing HR; #*p* < 0.001 vs. control siRNAs transfected HL-1 cells, subjected to HR.

Since siRNA knockdown of p38MAPKα affected MK2 but not ATF2 phosphorylation, we included MK2-deficient mouse embryonic fibroblasts (MEFs) [23] in our analyses and exposed them to HR. As noticed previously in MK2-deficient mice [23] MEFs also expressed lower levels of p38MAPK protein compared to wild-type controls. However, p38MAPK and MK2 activation occurred normally during HR and the treatment with BIRB796 showed the expected decrease in their activities (Figure 2A). While we did not observe a difference in basal ROS production between wild-type and MK2 knockout cells, the increase in HR-induced ROS levels was significantly lower in MK2-deficient cells (Figure 2B, C). Consistent with a role of MK2 downstream of p38MAPK, ROS production could also be decreased in wild-type cells through the application of BIRB796 but not in MK2-deficient cells (Figure 2B, C). However, application of the antioxidant N-acetyl-cysteine (NAC) was more potent in decreasing ROS levels (Figure 2B, C), arguing for additional p38MAPKα/MK2-independent modes of regulation. To exclude the possibility that down-regulation of p38MAPKα rather than the knockout of MK2 caused decreased ROS levels, we carried out the conditional knockdown of MK2 in HL-1 cells. While we were able to efficiently decrease MK2 protein levels in these cells, p38MAPK expression remained unaffected (Figure 2D). Knockdown of MK2 inevitably reduced phosphorylation of the MK2 substrate HSP25 under HR (Figure 2D). Again we observed decreased ROS levels as a result of MK2 knockdown, further supporting that the regulation of ROS via p38MAPK proceeded through MK2 (Figure 2E, F).

### p38MAPK inhibition protects from ischemia/reperfusion injury (IRI)

**Figure 3:**
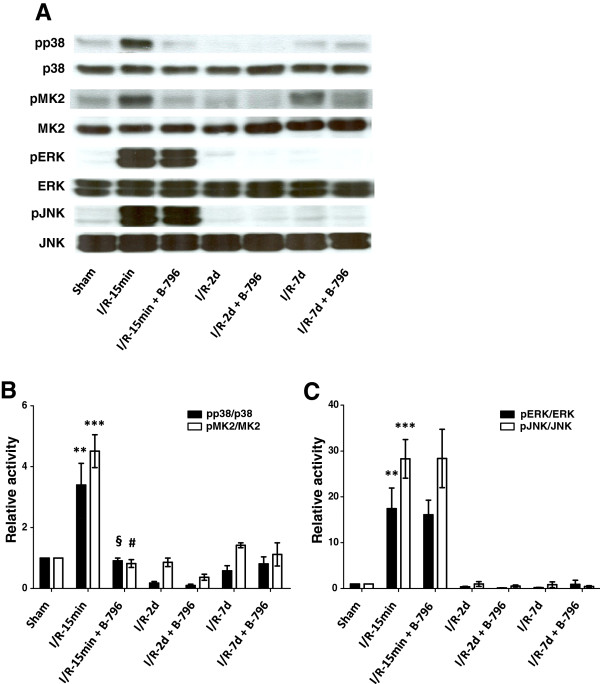
**Effect of p38MAPK (p38) inhibition on intracellular signaling following IR.** Rats were pretreated with the carrier DMSO or BIRB796 (B-796) (5 mg/kg BW) for 1 hour and subjected to 1 hour of renal ischemia followed by different time points of reperfusion (15 min, 2 days, 7 days). Kidneys were harvested at given time points of reperfusion and total tissue lysates were used to determine activation pattern of MAPKs (p38MAPK, JNK, ERK) and the downstream target of p38MAPK (MK2) by phosphorylation specific antibodies. A representative immunoblot **(A)** and summary graphs **(B, C)** are shown. Results are given as mean ± SEM (n = 3). ** *p* < 0.01, ***p < 0.001 vs. sham-operated group; §*p* < 0.01, #*p* < 0.001 vs. IR-15 min group.

To test whether p38MAPK inhibition may provide a clinically feasible approach for the prevention of IRI we used kidney clamping in the rat, a model that has been extensively characterized and allows monitoring of the damage progression by using reliable markers [1,24,25]. In our *in vitro* and *in vivo* models studied previously we had consistently observed maximum signaling activity between 10 and 15 min after reperfusion and reoxygenation, respectively [14] (and data not shown), and we thus again performed a first analysis at this time point. Clamping of the renal artery for 1 hour followed by 15 min of reperfusion resulted in a pronounced activation of p38MAPK (Figure 3A, B). The overall pattern of p38MAPK activation is comparable with the one observed in HL-1 cells under HR and in the previously published heterotopic heart transplant model [14]. Intraperitoneal application of BIRB796 (5 mg/kg BW), one hour before clamping, reduced p38MAPK activity to the background levels observed in sham-operated animals (Figure 3A, B). The changes in p38MAPK activity were also mirrored in the phosphorylation of its substrate MK2 (Figure 3A, B). No effect of the p38MAPK inhibitor on the activation of the related MAPKs ERK and JNK was observed (Figure 3A, C), indicating that within this group of kinases the expected specificity was achieved.

**Figure 4:**
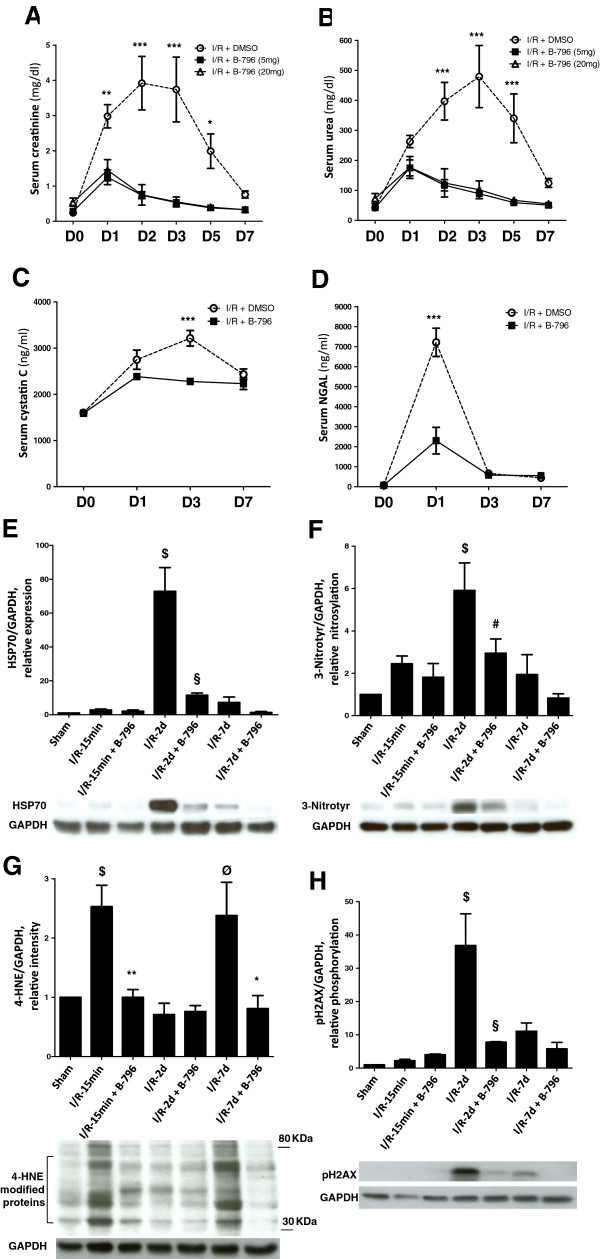
**p38MAPK (p38) inhibition prevents ischemia/reperfusion-induced increase in the serum levels of kidney function markers and oxidative stress indicators.** Serum levels of creatinine **(A)** and urea **(B)** were measured on indicated days (D0 to D7) following IR in rats pretreated with either BIRB796 (B-796) at two different doses (5 mg or 20 mg/kg BW) or vehicle (DMSO) only. Day 0 represents measurements before ischemia in every group. Likewise, serum cystatin C **(C)** and NGAL **(D)** levels were measured on indicated days of reperfusion in another set of experiments where rats were pretreated with either BIRB796 (B-796) (5 mg/kg BW) or DMSO only. Results are given as mean ± SEM (n = 4-7). *p < 0.05, **p < 0.01 ***p < 0.001, difference between DMSO- and BIRB796-treated groups at the given time points. **(E-H)** Rats were pretreated with BIRB796 (B-796) (5 mg/kg BW) for 1 hour and subjected to 1 hour of renal ischemia followed by different time points of reperfusion (15 min, 2 days, 7 days). Kidneys were harvested at given time points of reperfusion and total tissue lysates were used to determine the expression level of HSP70 **(E)**, the abundance of 3-nitrotyrosine (3-Nitrotyr) **(F)** and 4-HNE modified proteins **(G)**, and the phosphorylation of H2AX **(H)**. Results are given as mean ± SEM (n = 3-4). $*p* < 0.01, Ø*p* < 0.05 vs. sham-operated group, **p < 0.01 vs. IR-15 min group, §*p* < 0.01, #*p* < 0.05 vs. IR-2d group, *p < 0.05 vs. IR-7d group.

To monitor kidney function serum creatinine and urea levels were monitored over a period of seven days following renal IR. While setting up the model system we had chosen an ischemia time, which resulted in a pronounced deterioration of kidney function that was largely recoverable within a seven days period as based on the parameters chosen here. As shown in Figure 4A and B, serum creatinine and urea increased over two days of reperfusion to peak at day 3 in animals treated with DMSO (carrier). Treatment with the p38MAPK inhibitor prevented this increase, suggesting that functional damage to the kidney had been averted. Since the use of both biomarkers has been reviewed critically [26], we also included in our analyses two additional recently characterized marker proteins cystatin C [27] and NGAL [26,28]. The increase in cystatin C serum levels was significantly less pronounced in p38MAPK inhibitor-treated animals (Figure 4C). Similar results were obtained with NGAL (Figure 4D). Taken together, all markers tested responded to ischemia/reperfusion with the expected increase, which was sensitive to the inhibition by BIRB796. In a recent report HSP70 was suggested as an early and sensitive biomarker of acute kidney injury (AKI), suitable also for monitoring of renoprotective strategies [29]. IR caused a pronounced increase in HSP70 protein levels by day 2, while application of BIRB796 dramatically decreased the effect on HSP70 expression, indicating reduced incidence of AKI with this treatment (Figure 4E).

**Figure 5:**
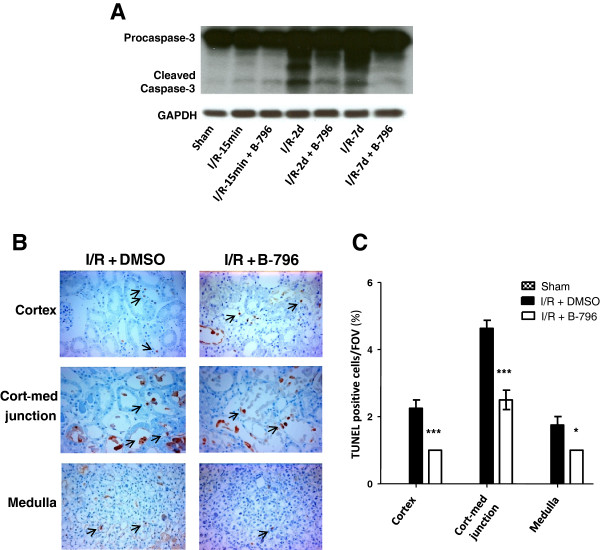
**p38MAPK (p38) inhibition prevents ischemia/reperfusion-induced apoptosis of tubular cells.** Rats were pretreated with the carrier DMSO or BIRB796 (B-796) (5 mg/kg BW) for 1 hour and subjected to 1 hour of renal ischemia followed by different time points of reperfusion (15 min, 2 days, 7 days). Kidneys were harvested at given time points of reperfusion and total tissue lysates were used to determine activation of caspase-3. A representative immunoblot is shown **(A)**. IR-induced tubular cell death was assessed by TdT-mediated dUTP nick end labeling (TUNEL) staining at day 2 of reperfusion as described in Material and Methods. Representative images of the three regions of the kidney (cortex, corticomedullary junction and medulla) at 400x magnification and summary graph of the TUNEL positive cells are shown **(B-C)**. Arrows point to the apoptotic cells with condensed nuclear material. Results are given as mean ± SEM (n = 4). *p < 0.05, ***p < 0.001 vs. vehicle-treated IR group.

While various methods can be used to measure ROS directly in isolated cells and tissues, no robust methods are available for *in vivo* application. Thus the use of markers for the oxidation of lipids, nucleic acids or proteins provides indirect means to gain insight into the generation of ROS [30]. First, cell lysates were probed with antibodies directed against 3-nitrotyrosine, a marker for oxidized proteins. The analysis of day 2 samples revealed increased levels of this redox modification, which were significantly reduced in inhibitor-treated animals (Figure 4F), indicating lower levels of ROS/NO. We also tested in the same samples a biomarker for oxidized lipids, 4-hydroxynonenal (4-HNE) [30]. As shown in panel G of Figure 4, lipid peroxidation was already visible at the earliest reperfusion time point analyzed and again at day 7, suggesting 4-HNE as an early marker of redox stress. In all cases signal intensity decreased to background levels in animals receiving the p38MAPK inhibitor. As a final parameter we also analyzed the phosphorylation modification of histone 2A family member X (H2AX), which serves as an indicator of endogenous oxidants [31]. As in the case of 3-nitrotyrosine, phosphorylation was most prominent on day 2 and again almost completely abolished by p38MAPK inhibition (Figure 4H). Together, these data demonstrate that application of BIRB796 prevents functional impairment usually associated with IR in the model chosen here. Finally, we also analyzed the effects of p38MAPK inhibition on cell death in the kidney by analyzing the processing of inactive caspase-3 in total kidney lysates (Figure 5A) and by TUNEL staining (Figure 5B, C). Strikingly, p38MAPK inhibition strongly blocked caspase 3 activation (Figure 5A) and in inhibitor-treated animals a significant decrease in the number of apoptotic tubular cells, particularly in the corticomedullary region, was also observed (Figure 5B, C).

## Discussion

While excessive ROS production is responsible for the development of IRI, the use of antioxidants in the clinic has been faced with little success for its prevention [13,32]. Accumulating evidence suggests that signaling proteins may be targeted to modulate mitochondrial processes including ROS production [13,14,32,33]. In our work we focused on p38MAPK, which gets activated during IR and for which potent low molecular weight inhibitors are available. This kinase has been implicated in the development of IRI [14,18-21], mainly through induction of cell death. However, while ROS have been implicated in the activation of MAPKs [34], we show here that this early activation of p38MAPK during reperfusion actually works upstream of changes in cellular ROS levels. We firmly establish p38MAPK as inducer of cellular redox stress by performing siRNA-mediated knockdown of the predominantly expressed p38MAPKα isoform in HL-1 cells and provide evidence for a role of MK2 as a possible downstream effector in this process. Most importantly, we can show that p38MAPK is an important inducer of pro-oxidant stress *in vivo* and that inhibition of p38MAPK activation in a rat model of renal IRI prevented the functional deterioration caused by IR.

The development of strategies for the prevention of renal ischemia/reperfusion injury (IRI) is essential as this condition is one of the most common causes of acute renal failure resulting in increased morbidity and mortality [35]. In particular the early phase of reperfusion, when the major ROS release occurs, is critical for the further course of events. Once produced, ROS directly damage proteins, lipids and nucleic acids [34] and they trigger various forms of cell death, resulting in the release of endogenous ligands (damage-associated molecular patterns, DAMPs) that activate signaling pathways, including the stress kinases JNK and p38MAPK [36]. DAMP-activated Toll-like receptor 4 (TLR-4) signaling, leading to the production of ROS through NOX4, has been implicated in the apoptosis of post-hypoxic TLR4-expressing renal tubule epithelial cells (RTECs) [37]. Moreover, ROS themselves have been linked to the activation of MAPKs and cell injury [38]. One scheme involves apoptosis signal-regulating kinase 1 (ASK1) [39,40], from which the negative redox sensor thioredoxin dissociates, resulting in the formation of an active ASK1 complex after the recruitment of TNF receptor-associated factors 2 (TRAF2) and 6 (TRAF6) and the activation of Jun N-terminal kinase (JNK) [41] or p38MAPK [42]. Thus halting the early ROS production holds the promise to prevent or limit further damage amplification. Our findings suggest that preventing p38MAPK activation, which occurs early during reperfusion, may achieve this goal. We currently do not know what activates p38MAPK in this setting, whether this reflects DAMP signaling or is induced by a first wave of ROS production, which then is further amplified by p38MAPK activation. p38MAPK may be a highly suitable target for intervention as it is also involved in inflammation signaling [43], which additionally contributes to the development of IRI [1]. In this regard inhibiting p38MAPK may be superior to interfering with NFκB signaling, which efficiently blocked inflammation during intestinal ischemia/reperfusion but at the same time also caused severe damage to the reperfused mucosa due to the lack of NFκB survival activity [44].

p38MAPK and some of its upstream components have been implicated in the regulation of cellular stress-induced cell and organ damage. Cardioprotection during IR has been reported following the disruption of a single copy of the p38MAPKα gene [45]. Inhibition of the p38MAPK upstream kinase MAP3K TGFβ-activated kinase 1 (TAK1) protected against oxygen and glucose deprivation (OGD) in primary cortical neurons and reduced the infarct volume after middle cerebral artery occlusion *in vivo*[46]. However, only short term, but not prolonged inhibition of TAK1 was protective by inferring with the activation of p38MAPK and JNK and the formation of superoxide. In cultured cardiac myocytes the MAP2K MKK6 directly stimulated p38MAPK through phosphorylation and activated p38MAPK promoted cell survival, while activation by the related MKK3 resulted in death [47,48]. Consequently, MKK6 transgenic mouse hearts were protected against IR through a mechanism which involved upregulation of the small heat shock protein alpha B-crystallin [49]. The fact that inhibition of stress kinase signaling may be protective in the setting of ischemia/reperfusion by preventing cell death has been pointed out before. Thus the cardioprotective action of Sirt1 during IR results from reducing the activation of JNK and p38 [21]. Similarly the protective effects of curcumin in left anterior descending coronary artery (LAD) occlusion goes along with the attenuation of p38 and JNK activity [20]. The protective effect was further enhanced by simultaneous activation of several prosurvival kinases [20]. Direct p38MAPK inhibition decreased cardiomyocyte apoptosis and helped to maintain cardiac function in the Langendorff-perfused rabbit heart [50]. In our work [14] we previously have obtained evidence that p38MAPK signaling is activated during IR and pilot studies in cardiomyocytes undergoing hypoxia/reoxygenation showed that p38MAPK inhibition not only reduces ROS levels but also cell death [14]. However, none of the published reports placed p38MAPK above ROS production, thus making it a suitable target for the prevention of IRI, which cannot be prevented by antioxidants.

How does the inhibition of p38MAPK prevent IRI? Most strikingly, in our experiments protection by BIRB796 was afforded under severe ischemic conditions (ischemia time of 1 hour), as documented by the established functional markers creatinine and urea. While serum creatinine and urea provide measures of kidney function, increase in Hsp70 [29] and in particular NGAL [51,52] are indicators of renal epithelial injury [53]. These findings are also corroborated by the observed protective effect of p38MAPK inhibition on the survival of tubular epithelial cells (Figure 5B, C). Apoptosis is usually caused by the acute ischemic or nephrotoxic events that are not severe enough to induce tubular necrosis and result in a different degree of impairment and acute renal failure (ARF). Therapeutic interventions that inhibit apoptosis of renal tubular cells have the potential of minimizing deterioration of renal function.

Biochemical analyses also suggested the prevention of oxidative damage in the tissues of p38MAPK inhibitor-treated animals (Figure 4). Damage caused by IR has been linked predominantly to mitochondrial ROS, although there is evidence for the involvement of non-mitochondrial ROS sources [54]. In our *in vitro* experiments we used MitoTracker Red CM-H2XRos a ROS-sensitive dye, which should specifically detect ROS located in the mitochondria. Since increased ROS levels and cell death depended on p38MAPK activity and the antioxidant N-acetyl cysteine (NAC) prevented apoptosis [14], p38MAPK may cause damage by increasing mitochondrial ROS levels. The highly reactive and short-lived nature of ROS makes it difficult to measure them *in vivo* and surrogate markers are commonly used. Nitration of tyrosine residues by peroxynitrites, generated by the rapid interaction of superoxides with nitric oxide (NO), leads to the formation of nitrotyrosine [55]. Our data showed increase in 3-nitrotyrosine formation at day 2 of reperfusion, which was significantly reduced following the treatment with BIRB796 (Figure 4F). These findings could be confirmed by using 4-HNE as a marker for lipid peroxidation (Figure 4G). The reduction in oxidative damage following p38MAPK inhibitor treatment also correlated with the reduced functional impairment of the kidney following IR, supporting a direct role for ROS in causing damage to the kidney. Similar results were obtained using a third marker, the phosphorylation modification of H2AX (Figure 4H).

It is still an open question how the damage signal is propagated downstream of p38MAPK. Our work suggested that this at least in part may involve MK2. MK2-deficient hearts subjected to 30 min ischemia followed by 2 hours of reperfusion in the Langendorff model were resistant to myocardial IRI and also showed a diminished number of apoptotic cardiomyocytes [56]. This view was contrasted by findings obtained in a later study [23], which used a related approach. Similar infarct sizes were observed in wild-type and MK2 deficient hearts, however, these could be decreased by the p38MAPK inhibitor SB203580 [23], suggesting p38MAPK targets outside of MK2. Protection again was observed in a model of cerebral ischemic injury in MK2-deficient mice [48]. Future work will have to chart possible signaling connections between MK2 and mitochondrial ROS production.

## Conclusions

ROS are causal for development of ischemia/reperfusion injury and consequential organ dysfunction or failure. Here we demonstrate for the first time that inhibition of p38MAPK prevents the early ischemia/reperfusion-associated decline in organ function, which may halt further deterioration. Our data hint that reducing redox stress and cell death may be the underlying cause. p38MAPK inhibition may thus be an alternative to antioxidants, which failed in the clinic for the prevention of redox stress-associated organ damage.

## Methods

### Cell culture and hypoxia/reoxygenation (HR) induction

The HL-1 cardiomyocyte cell line has been derived from AT-1 mouse atrial myocytes, obtained from transgenic mice expressing SV40 large T antigen under the control of atrial natriuretic factor (ANF) promoter [57,58]. Cells were maintained in Claycomb medium (Sigma Aldrich, Schnelldorf, Germany) supplemented with 10% fetal calf serum (FCS) (PAA Laboratories, Pasching, Austria), penicillin (100 U/ml) (PAA Laboratories, Pasching, Austria), streptomycin (100 μg/ml) (PAA Laboratories, Pasching, Austria), 0.1 mM norepinephrine (Sigma Aldrich, St. Louis, MO, USA) and 2 mM L-glutamine (GIBCO Invitrogen, Grand Island, NY, USA), as described previously [57]. Murine embryonic fibroblasts (MEFs), isolated from WT and MK2 deficient mice [59] (provided by Matthias Gaestel, Hannover, Germany), were cultivated in DMEM (PAA Laboratories, Pasching, Austria) containing 10% FCS, 2 mM L-glutamine, penicillin (100 U/ml) and streptomycin (100 μg/ml). Cells were subjected to hypoxia/reperfusion (HR) as described previously [14]. Briefly, for induction of hypoxia (H) cells were maintained in starvation medium (DMEM or Claycomb medium, containing 0.05% FCS) and exposed to 0.5% O_2_ at 37°C for 1 or 6 hours using a Modular Incubator Chamber (Billups-Rothenberg, Del Mar, CA, USA). For subsequent reoxygenation (R), starvation medium was replaced by standard culture medium. BIRB796, a highly potent ATP-competitive type II inhibitor of p38MAPK [60] (commercially obtained from Axon MedChem, Groningen, The Netherlands or kindly provided by Boehringer Ingelheim Pharmaceuticals, Ridgefield, CT, USA), was used in all *in vitro* and *in vivo* experiments described here.

### Immunoblotting

Whole cell and tissue lysates were prepared using ice cold NP40 lysis buffer (25 mM TRIZMA base, 150 mM NaCl, 10 mM Na_4_P_2_O_7_, 25 mM β-glycero-phosphate, 10% glycerol, 0.75% NP-40, 25 mM NaF, pH 7.2) and RIPA lysis buffer (1% NP-40, 1% CHAPS, 0.1% SDS, 0.15 M NaCl, 10 mM Na-phosphate, 2 mM EDTA, 50 mM NaF, pH 7.2), respectively, containing 1:100 protease inhibitor cocktail set-I (Calbiochem, Darmstadt, Germany) and Na-orthovanadate (0.2 mM). Protein content was determined by using Bio-Rad DC protein assay kit (Bio-Rad, Hercules, CA, USA). Immunoblotting was performed as described previously [14,61]. Briefly, proteins were separated by SDS-PAGE and transferred to nitrocellulose membrane. The membranes were blocked in 5% skim milk powder (Fluka, Buchs, Switzerland), dissolved in TBST (50 mM TRIZMA base, 150 mM NaCl, pH 7.5 adjusted with HCl, 0.1% Tween-20), for one hour at room temperature and probed over night with appropriate primary antibodies, diluted in 5% BSA or skim milk powder as recommended by the provider, followed by incubation for one hour in HRP-conjugated secondary antibody, diluted in 5% skim milk. Primary antibodies against phospho-p38MAPK (9211), p38MAPK (9212), phospho-MAPKAP kinase 2 (3044), MAPKAP kinase 2 (3042), phospho-ATF2 (9221), Caspase-3 (9662), phospho-H2AX and phospho-HSP25 (2401) were obtained from Cell Signaling Technology, Boston, MA, p38MAPKα (sc-535), HSP70 (sc-66048), ATF2 (sc-187), phospho-ERK (sc-16982R), ERK1 (sc-94) and JNK (sc-571) from Santa Cruz Biotechnology, Santa Cruz, CA, phospho-JNK (AF1205) from R&D systems, Minneapolis, MN, USA, HSP25 (SPA-801) from Enzo Life Sciences, Lausen Switzerland, 3-Nitrotyrosine (ab52309) from Abcam, Cambridge, UK, GAPDH (AM4300) from Ambion, Grand Island, NY, α-tubulin (T5168) from Sigma Aldrich Dorset, UK and 4-hydroxy-2-nonenal (4-HNE) from Japan Institute for the Control of Aging. Antibodies were visualized by ECL western blot detection reagents (Amersham, Buckinghamshire, UK), quantified by densitometric scanning using the Image J program (NIH, Bethesda, MD) and normalized against loading controls.

### siRNA transfection

Cells were transfected one day after seeding in a 6 well plate using DharmaFECT-1 (Dharmacon RNA technologies, Lafayette, CO, USA). ON TARGETplus SMARTpool small interfering RNAs (siRNAs) for mouse p38MAPKα and MAPKAP kinase 2 (MK2) were obtained from Thermo Scientific (Lafayette, CO, USA). The effect of siRNA knockdown on endogenous p38MAPKα and MK2 expression, respectively, was confirmed by immunoblotting 72 hours post transfection. For negative controls, microarray-tested siGENOME Non-Targeting siRNA 2 (Thermo Scientific, Lafayette, CO, USA) was used, which according to company information possesses at least four mismatches to any human, mouse, or rat gene. 48 hours after transfection, cells were set up in parallel to study the effect of p38MAPKα and MK2 knockdown on p38MAPK downstream signaling and mitochondrial ROS production. 72 hours after transfection cells were used in experiments.

### RNA isolation and cDNA synthesis

Total RNA from tissues and HL-1 cells was isolated using the Macherey-Nagel NucleoSpin RNA II Kit (Macherey-Nagel, Dueren, Germany) according to the manufacturer’s instructions, which included a DNAse-I digest to avoid contamination with genomic DNA. RNA integrity and quality was verified by agarose gel-electrophoresis and spectrophotometrically, respectively. For cDNA synthesis 1-2 μg of total RNA was reverse transcribed in a 30 μl reaction volume using oligo(dT) primer and the RevertAid™ First Strand cDNA Synthesis Kit (Fermentas GmbH, St. Leon-Rot, Germany).

### Quantitative real time (qRT)-PCR

The following primer pairs specific for murine isoforms of p38MAPK (α, β, γ, δ) and the housekeeping gene RPS29 were designed; p38MAPKα forward: 5′-GCCTGTTGCTGACCCTTATGACC-3′, p38MAPKα reverse: 5′-GGGGTGGTGGCACAAAGCTGAT-3′, p38MAPKβ forward: 5′-AGCCCTATGATGAAAGTGTTGAGGC-3′, p38MAPKβ reverse: 5′-TCAATTTCATGGGTGCCAGGGAGC-3′, p38MAPKγ forward: 5′-CTGAGTTTGTTCAGAAGCTACAGAG-3′, p38MAPKγ reverse: 5′-ACAGCCTGAGGGCTTGCGTTG-3′, p38MAPKδ forward: 5′-CCCCAAGAAGGATTTC ACACAGC-3′ , p38MAPKδ reverse: 5′-GTGTTGTTTCCATTCGTCCACGC-3′, RPS29 forward: 5′-GCTCTACTGGAGTCACCCACGGAA-3′, RPS29 reverse: 5′-ACTGGCACATGTTCAGCCCGTA-3′. The primers were taken from regions with the lowest sequence homology between the isoforms with at least 3 bases at the 3′-end of the primer being absolute (100%) specific for the respective isoform. The optimal annealing temperature for each primer pair was determined experimentally using conventional temperature gradient PCR (Taq DNA Polymerase, Life Technologies, Paisley, UK) from mouse heart cDNA as template. The annealing temperature gradient was from 54°C to 68°C. PCR products were visualized by ethidium bromide staining under UV-light following separation on 1.4% agarose-gels. The determined annealing temperature yielding only the specific PCR product of the expected size was used for qRT-PCR analysis. Furthermore, melting curve analysis following qRT-PCR revealed one specific peak for each primer pair (data not shown). Specificity of primer pairs was confirmed by BLAST (NCBI), and possible dimer formation was analyzed using FastPCR [62]. All primer pairs were intron-spanning. Quantitative RT-PCR reactions were performed in triplicates in a 25 μl volume containing 5 μl of the 1:5 diluted cDNA, 0.5 μM of each primer (Biomers, Ulm, Germany), and 5 μl five-fold iQ™ SYBR® Green Supermix (Bio-Rad Laboratories GmbH, Munich, Germany) on a Bio-Rad iQ5 Cycler (Bio-Rad Laboratories GmbH, Munich, Germany). After each run a melting curve analysis was carried out to confirm the specificity of the PCR products. Data were normalized to the housekeeping gene RPS29 and the untreated controls using the in-built software (normalized fold expression).

### Measurement of ROS levels by fluorescent imaging

40-60 × 10^3^ cells were placed in fibronectin/gelatin-coated Lab-Tek chambered cover glass (Nalge Nunc, Rochester, NY), and pretreated with either the carrier DMSO, BIRB796 or N-acetyl cysteine for 1 hour and then subjected to a hypoxia/reperfusion protocol: hypoxia (1 or 6 hours, 0.5% O_2_, 37°C, 0.05% FCS DMEM or Claycomb medium) and reoxygenation (2 or 15 min, normoxic atmosphere, 37°C, DMEM or Claycomb medium). For the staining procedure, the cells were incubated with MitoTracker Red CM-H2XRos (0.2 μM; Invitrogen Molecular Probes, Eugene, OR, USA) at 37°C for one hour when hypoxia time of one hour was used or for 15 min, when the hypoxia time was 6 hours followed by 15 min of reperfusion (during reperfusion). Digital images were taken using an Olympus IX-70 inverted microscope (Olympus America, Melville, NY, USA) with an Olympus 40 × water immersion objective (numerical aperture 0.8) and an Olympus U-RFL-T mercury-vapor lamp. Images were acquired using a Kappa ACC1 camera and Kappa ImageBase software (Kappa Opto-electronics, Gleichen, Germany). For MitoTracker Red CM-H_2_XRos a 568 nm-filter was used. Grey values were measured using Scion Image software for Windows. For every experimental condition grey values from 80-100 cells were averaged.

### Rat kidney clamping

Male Lewis rats weighing 220-250 g were obtained from Charles River, Germany and kept with unlimited access to water and standard laboratory chow in agreement with local guidelines and the Austrian Animal Care Law. All experiments were approved by the Austrian Ministry of Education, Science and Culture and were performed in accordance with national animal protection guidelines. Rats were anaesthetized by intramuscular injection of ketamine (100 mg/Kg BW) and xylazine (10 mg/kg BW) (Graeub Veterinary Products, Bern, Switzerland). A middle incision was made to expose the abdominal cavity and after right side nephrectomy the left renal artery was identified and liberated by blunt dissection. Renal artery was clamped (1 hour) using micro serrefine clamps (FST# 18055-01; Fine Scientific Tools, Heidelberg, Germany) to induce ischemia, followed by different times of reperfusion (15 min, 2 days, and 7 days). Renal occlusion was macroscopically verified by the change in color of the kidneys to pale and reperfusion by a blush appearance of the kidney. DMSO/BIRB796 (5 or 20 mg/kg BW) was applied intraperitoneally one hour prior to vessel clamping. Surgery was performed at room temperature; however, soon after kidney clamping the animal was placed on the heating pad (37°C) and kept there during ischemia/clamping (1 hour) and early reperfusion until the animal recovered from anesthesia. At the given time points of reperfusion the animals were sacrificed and kidney tissues were harvested and stored in neutrally buffered formaldehyde (4.5%) and liquid N_2_ for tissue histology and biochemical analyses, respectively. The sham-operated animals underwent the surgical procedure that was identical to the ischemic rats except that the clamps were not applied.

### Assessment of renal function

Renal function was assessed by serum creatinine, urea, cystatin C and NGAL measurement. Blood samples (0.2 - 0.4 ml) were taken from the tail vein before ischemia as well as at various time points after reperfusion and centrifuged (10800 × g for 4 min) to isolate serum. Serum creatinine and urea were routinely measured at the Central Institute for Medical and Chemical Laboratory Diagnostics (ZIMCL) of the Innsbruck Medical University (IMU). Serum cystatin C and NGAL were measured using ELISA kits (BioVendor, Brno, Czech Republic).

### TUNEL staining (terminal deoxynucleotidyl transferase (TdT)-mediated dUTP nick end labeling)

Kidney samples were fixed and collected in 4.5% neutrally buffered formaldehyde at room temperature until further processing. Dehydration, paraffin embedding and preparation of slides were done following standard histology procedures. For the assessment of apoptotic cells tissue sections were stained with the *In Situ* Cell Death Detection Kit (Roche Inc., Mannheim, Germany). For antigen retrieval the slides were pretreated in citrate buffer (pH 6) in the microwave at 750 watt for 15 min and endogenous peroxidase was blocked by incubation in 5% H_2_O_2_ in methanol for 15 min. Afterwards, the TUNEL reaction mixture was prepared freshly and the slides were incubated for 45 min in a humidified chamber. Slides were counterstained with hematoxylin-eosine (HE) and subsequently analyzed under light microscope. Each histological section was divided into 3 parts (cortex, corticomedullar junction and medulla). The results were quantified by counting the number of positively stained cells per 5 high-power fields (HPF) at 400 × magnification for each of the 3 areas and given as percentage.

### Statistical analyses

All numerical data are expressed as mean values ± SEM. The experimental groups were compared using one way or two way ANOVA followed by Bonferoni post hoc test. A p value of <0.05 was considered to be statistically significant.

## Abbreviations

IRI: Ischemia/reperfusion injury; ROS: Reactive oxygen species; MK2: MAPKAP kinase 2; HR: Hypoxia/reoxygenation; IR: Ischemia/reperfusion; MAPK: Mitogen activated protein kinase; DAMPs: Damage-associated molecular patterns; NAC: N-acetyl cysteine; B-796: BIRB796 (p38MAPK inhibitor).

## Competing interests

The authors declare no competing interests.

## Authors’ contributions

MIA, DD, GB, JT contributed to the design of the study. MIA, ME, CW, MH, HS, MHE, SS, KK, ME, SK, AS, CS, SV, RS performed the experiments and analysed the data. MIA compiled the data and prepared a first manuscript draft, MIA, DD, JT contributed to the writing of the manuscript. All authors read and approved the final version of this manuscript.
